# The Role of Coronary Computed Tomography Angiography in the Diagnosis, Risk Stratification, and Management of Patients with Diabetes and Chest Pain

**DOI:** 10.31083/j.rcm2512442

**Published:** 2024-12-17

**Authors:** Willem R. van de Vijver, Jasper Hennecken, Ioannis Lagogiannis, Candelas Pérez del Villar, Cristian Herrera, Philippe C Douek, Amit Segev, G. Kees Hovingh, Ivana Išgum, Michiel M. Winter, R. Nils Planken, Bimmer E.P.M. Claessen

**Affiliations:** ^1^Department of Cardiology, Heart Center, Amsterdam University Medical Centers, University of Amsterdam, 1105 AZ Amsterdam, The Netherlands; ^2^Cardiology Centers of the Netherlands, 3544 AD Utrecht, The Netherlands; ^3^Department of Biomedical Engineering and Physics, Amsterdam University Medical Centers, University of Amsterdam, 1105 AZ Amsterdam, The Netherlands; ^4^Informatics Institute, Faculty of Science, University of Amsterdam, 1098 XH Amsterdam, The Netherlands; ^5^Department of Cardiology, University Hospital of Salamanca, 37007 Salamanca, Spain; ^6^Instituto de Investigación Biomédica de Salamanca (IBSAL), 37007 Salamanca, Spain; ^7^CIBER de Enfermedades Cardiovasculares, Instituto de Salud Carlos III, 28029 Madrid, Spain; ^8^University of Lyon, INSA-Lyon, Claude Bernard Lyon 1 University, UJM-Saint Etienne, CNRS, Inserm, 69621 Villeurbanne, France; ^9^Hospices Civils de Lyon, Department of Radiology, Hopital Cardiologique Louis Pradel, 69500 Bron, France; ^10^Department of Cardiology, Leviev Heart Center, Chaim Sheba Medical Center, 52621 Tel Hashomer, Israel; ^11^The Faculty of Medicine, Tel Aviv University, 69978 Tel Aviv, Israel; ^12^Department of Vascular Medicine, Amsterdam University Medical Centers, University of Amsterdam, 1105 AZ Amsterdam, The Netherlands; ^13^Department of Radiology and Nuclear Medicine, Amsterdam University Medical Centers, University of Amsterdam, 1105 AZ Amsterdam, The Netherlands

**Keywords:** coronary artery disease, coronary computed tomography angiography, diabetes mellitus

## Abstract

Coronary artery disease (CAD) affects over 200 million individuals globally, accounting for approximately 9 million deaths annually. Patients living with diabetes mellitus exhibit an up to fourfold increased risk of developing CAD compared to individuals without diabetes. Furthermore, CAD is responsible for 40 to 80 percent of the observed mortality rates among patients with type 2 diabetes. Patients with diabetes typically present with non-specific clinical complaints in the setting of myocardial ischemia, and as such, it is critical to select appropriate diagnostic tests to identify those at risk for major adverse cardiac events (MACEs) and for determining optimal management strategies. Studies indicate that patients with diabetes often exhibit more advanced atherosclerosis, a higher calcified plaque burden, and smaller epicardial vessels. The diagnostic performance of coronary computed tomographic angiography (CCTA) in identifying significant stenosis is well-established, and as such, CCTA has been incorporated into current clinical guidelines. However, the predictive accuracy of obstructive CAD in patients with diabetes has been less extensively characterized. CCTA provides detailed insights into coronary anatomy, plaque burden, epicardial vessel stenosis, high-risk plaque features, and other features associated with a higher incidence of MACEs. Recent evidence supports the efficacy of CCTA in diagnosing CAD and improving patient outcomes, leading to its recommendation as a primary diagnostic tool for stable angina and risk stratification. However, its specific benefits in patients with diabetes require further elucidation. This review examines several key aspects of the utility of CCTA in patients with diabetes: (i) the diagnostic accuracy of CCTA in detecting obstructive CAD, (ii) the effect of CCTA as a first-line test for individualized risk stratification for cardiovascular outcomes, (iii) its role in guiding therapeutic management, and (iv) future perspectives in risk stratification and the role of artificial intelligence.

## 1. Introduction

Coronary artery disease (CAD) remains the foremost cause of mortality globally, impacting over 200 million individuals in 2019, and is responsible for more than 9 million deaths annually [1, 2]. Diabetes mellitus (DM) is a significant risk factor for CAD, with evidence suggesting that patients with DM have up to a fourfold increased risk of developing CAD compared to patients without DM [3, 4]. Moreover, CAD accounts for 40–80% of deaths in patients with type 2 DM (T2DM) [5]. The International Diabetes Federation estimates a staggering 537 million adults live with DM worldwide, which is expected to rise to 643 million by 2030 [6]. Additionally, managing patients with DM presents unique challenges, particularly regarding the optimal evaluation and management of stable CAD. Numerous tests are available to assess ischemia in patients who present with symptoms indicative of stable obstructive CAD. While invasive coronary angiography (ICA) remains the gold standard for diagnosing CAD, it is not without significant risks. These include rare but serious procedure-related complications, considerable costs, logistical challenges, radiation exposure to both patients and physicians and patient discomfort. These inherent risks, largely due to the invasive nature of ICA, have spurred the development and use of noninvasive testing options.

Noninvasive assessments can be broadly divided into anatomical and functional imaging. Functional tests, such as stress electrocardiography, stress echocardiography, single-photon emission computed tomography (SPECT), stress positron emission tomography (PET), and stress cardiac magnetic resonance (CMR), each have their own diagnostic accuracy, advantages, and limitations. However, a key limitation of these functional methods is their ability to detect only ischemia resulting from atherosclerosis. This means that a negative result does not exclude the presence of non-flow-limiting atherosclerotic disease. In contrast, coronary computed tomography angiography (CCTA) offers detailed anatomical insights, allowing for quantitative and qualitative assessments of atherosclerotic plaques. This capability enables the identification and diagnosis of lower-grade stenoses that could be missed by functional testing, leading to more CAD diagnoses [7, 8]. This can potentially lead to a reduction in major adverse cardiac events (MACEs) through earlier initiation of preventive therapies aimed at more rigorous treatment targets in patients with DM and stable CAD [7, 8].

The diagnostic accuracy of CCTA in detecting obstructive CAD has been well-validated in multiple prospective studies [9, 10]. These studies have demonstrated high sensitivity but relatively low specificity, which makes it particularly valuable for ruling out CAD. Due to these attributes, CCTA is recommended as a primary diagnostic test for most patients presenting with stable chest pain, as endorsed by the European Society of Cardiology (ESC) and the American College of Cardiology (ACC)/American Heart Association (AHA) guidelines [11, 12]. Additionally, advancements in computed tomography-derived fractional flow reserve (CT-FFR) and stress myocardial perfusion computed tomography (CT) currently allow for functional evaluations, which are particularly useful in intermediate stenosis cases [13]. Furthermore, combining PET and CCTA in a single test offers functional and anatomical information [14].

CCTA allows for a thorough assessment of the coronary anatomy, including the presence of epicardial vessel stenosis and quantification of the total plaque burden and characteristics. These parameters are associated with incident MACEs, such as cardiac death, myocardial infarction, and the eventual need for revascularization [15, 16]. Based on previous clinical studies, the burden and morphology of plaques in patients with and without DM were shown to differ significantly [17, 18, 19].

With the emergence of CCTA as the preferred first-line test for evaluating chest pain, it is crucial to carefully consider its appropriateness in patients with DM, who have a higher risk of CAD and, therefore, potentially less favorable test results. Thus, this review assesses several key aspects of utilizing CCTA in patients with DM: (i) the diagnostic accuracy of CCTA in detecting obstructive CAD, (ii) the effect of CCTA as a first-line test for individualized risk stratification for cardiovascular outcomes, (iii) its role in guiding therapeutic management, and (iv) future perspectives in risk stratification and the role of artificial intelligence. This comprehensive review aims to delineate the specific benefits and limitations of CCTA in patients with DM. Furthermore, we cover several novel applications of CCTA, including the characterization of plaque characteristics and the implementation of artificial intelligence.

## 2. Diagnostic Accuracy of CCTA in Diagnosing Obstructive CAD in Patients with DM

The diagnostic accuracy of CCTA for detecting obstructive CAD may differ between patients with and without DM for several reasons. Firstly, the pre-test probability is higher in patients with DM [20]. Diagnostic accuracy has been shown to be lower in patients with a high likelihood of CAD, as the specificity of CCTA is relatively low [21, 22]. Secondly, the characteristics of atherosclerotic CAD in patients with DM differ from those of patients without. This has been described by Kip and colleagues (1996) [19], who studied the differences in invasive angiographic characteristics between patients in a multicenter registry with and without DM and found that the atherosclerotic disease of the epicardial vessels was more extensive and diffuse in patients with DM. Several other studies have reported a higher plaque burden and more calcification of atherosclerotic plaques in patients with DM [17, 18, 23]. The presence of calcified lesions is associated with the occurrence of blooming artifacts on CCTA, which can cause overestimations of lumen narrowing. Multiple studies have observed a reduction in specificity as the coronary artery calcium score (CACS) increases, which indicates a calcified plaque burden [24, 25, 26, 27]. A meta-analysis conducted by Abdulla and colleagues (2012) [24], including 1634 patients, demonstrated a decrease in specificity in patients with a CACS greater than 400 Agatston units (AU) (42% [95% confidence interval (CI) 28–56%]) compared to those with a CACS less than 100 AU (88.5% [95% CI 81–91.5%]).

We identified four studies evaluating the diagnostic accuracy of CCTA for detecting obstructive CAD in patients with DM (Table 1, Ref. [28, 29, 30, 31]). One study assessed diagnostic accuracy in 30 patients with chest pain and DM. The study cohort had a mean age of 62 years, and 87% of the participants were male. The sensitivity and specificity of CCTA were 81% and 82%, respectively, using ICA as the gold standard for detecting anatomical stenosis greater than 50%. Notably, 14% of the analyzed segments were deemed uninterpretable on the CCTA [28]. One of the remaining three studies reported lower diagnostic accuracy using 64-slice CT on patients with DM than those without [29]. In this study, 105 patients with DM and 105 without were referred for ICA due to suspected CAD and underwent CCTA before ICA. The pre-test likelihood of CAD was similar between the two groups (51% for patients with DM *vs*. 52% for patients without DM). Patients with DM had a significantly higher CACS (479 ± 492 *vs*. 356 ± 367, *p* = 0.01). Additionally, DM patients had more segments with artifacts (81 *vs*. 39, *p* < 0.0001) and significantly lower lumen diameters in most segments. CCTA demonstrated a sensitivity of 76%, specificity of 90%, positive predictive value (PPV) of 71%, negative predictive value (NPV) of 93%, and accuracy of 87% in patients with DM. For patients without DM, CCTA showed a sensitivity of 92%, specificity of 96%, PPV of 87%, NPV of 98%, and accuracy of 96%. All diagnostic parameters (sensitivity, specificity, NPV, PPV, and accuracy) were significantly higher in patients without DM (*p* = 0.001). In a patient-based model, CCTA identified 87 of 93 DM patients with significant stenoses at the ICA (sensitivity 94%). CCTA correctly ruled out significant CAD in three patients but falsely identified significant stenosis in nine cases (specificity 33%). Specificity, NPV, and accuracy were higher in patients without DM (83%, 79%, and 93%) than in those with DM (33%, 25%, and 86%) [29]. Two studies reported no statistically significant difference in diagnostic accuracy [30, 31]. The first retrospective analysis study included 22 patients with DM and 94 without who underwent CCTA and ICA for suspected CAD or progression of known CAD. There was no significant difference between the two groups in mean age (64.6 years *vs*. 64.2 years, respectively) or the proportion of males (77% *vs*. 64%). Patients with DM exhibited significantly higher risk factors for CAD than those without DM (3.4 *vs*. 2.4, *p* < 0.05). The CACS did not significantly differ between patients with and without DM (1090 ± 1278 *vs*. 798 ± 1033, respectively). In patients with DM, 46 (85%) of 54 significant stenoses (>50% luminal narrowing) in the coronary vessels were correctly detected by CCTA. Additionally, 215 (98%) of 219 segments without significant stenosis were correctly classified by CCTA. The sensitivity, specificity, PPV, and NPV were 85%, 98%, 92%, and 96%, respectively. In patients without DM, 244 (84%) of 290 significant stenoses were correctly detected by CCTA; of 893 segments without significant stenosis, 868 (97%) were correctly classified by CCTA. The sensitivity, specificity, positive, and negative predictive values were 84%, 97%, 91%, and 95%, respectively. The sensitivity, specificity and positive and negative predictive values did not differ significantly between patients with and without DM [30].

**Table 1.** **Studies evaluating the diagnostic accuracy of CCTA for detecting obstructive CAD in patients with diabetes mellitus**.

First author (year)	Sample size	Objective	Reference test	Population	Main results
Schuijf *et al*. (2004) [28]	30 patients with diabetes	To evaluate the diagnostic performance of CCTA in identifying coronary stenosis and evaluate ventricular functions	ICA; anatomical stenosis >50%	Patients referred for CCTA owing to the presence of stable chest pain	Sensitivity 81%, specificity 82%, PPV 62%, and NPV 92%
Andreini *et al*. (2010) [29]	Total: 210; 105 patients with diabetes	To compare the diagnostic performance of 64-slice MDCT between patients with and without diabetes	ICA; anatomical stenosis >50%	Patients referred for ICA owing to suspected CAD/inconclusive stress test	All diagnostic parameters were significantly higher in patients without diabetes (*p* = 0.001)
Burgstahler *et al*. (2007) [30]	Total: 116; 22 patients with diabetes	To compare the diagnostic performance of 16-slice MDCT between patients with and without diabetes	ICA; anatomical stenosis >50%	Patients referred for ICA owing to a suspicion of CAD or progression of previously diagnosed CAD	No significant difference in sensitivity, specificity, PPV, and NPV between patients with and without diabetes
Moon *et al*. (2013) [31]	Total: 240; 74 patients with diabetes	To compare the diagnostic performance of 64-slice MDCT between patients with and without diabetes	ICA; anatomical stenosis >50%	Patients referred for ICA owing to the presence of stable chest pain	No significant difference in sensitivity, specificity, PPV, NPV, and accuracy between patients with and without diabetes

Abbreviations: MDCT, multi-detector computed tomography; ICA, invasive coronary angiography; CCTA, coronary computed tomography angiography; CAD, coronary artery disease; PPV, positive predictive value; NPV, negative predictive value.

The second study involved 74 patients with DM and 166 patients without DM, all referred for ICA due to chest pain and who underwent CCTA within 30 days of ICA. Patients with DM had a mean age of 41.8 years, 54.1% were male, and a mean body mass index (BMI) of 23.5. Patients without DM had a mean age of 42.2 years, 64.5% were male, and a mean BMI of 23.8. Additional risk factors for CAD were not described. Patients with DM had a significantly higher mean coronary artery calcium (CAC) score than those without DM (658.6 *vs*. 426.4 AU). Of the 4064 coronary artery segments analyzed, both CCTA and ICA assessed 4062, and 706 were identified as significant ICA lesions. In patients with DM, 226 out of 1109 segments had significant stenotic lesions. On a per-segment basis, CCTA showed a sensitivity of 89.4%, specificity of 96.4%, PPV of 85.8%, NPV of 97.4%, and accuracy of 95.0%. In patients without DM, CCTA had a sensitivity of 83.8%, specificity of 97.6%, PPV of 88.0%, and NPV of 96.6%. There was no significant difference in these parameters between the two groups. Additionally, no significant difference was found on a per-patient basis. Furthermore, the CAC score did not significantly influence diagnostic accuracy [31]. Interestingly, the study by Andreini *et al*. (2010) [29] was the only one to report a significant difference in diagnostic accuracy; moreover, this was the only study in which a difference in CAC was observed.

A major limitation in these studies is the use of more than 50% anatomical stenosis as the reference standard, an arbitrary threshold that does not necessarily correlate with myocardial ischemia. In addition, the studies comparing diagnostic accuracy between patients with and without DM were limited by small sample sizes and selection bias, as they included only patients referred for ICA. This may reduce the generalizability of the findings, as diagnostic accuracy may be higher in patients referred for noninvasive testing, given the lower pre-test probability in this population. Moreover, the coronary CT angiography in these studies was performed using scanners that are now considered outdated. While these studies offer some useful insights, larger studies using modern imaging technologies are needed to fully assess the impact of DM on the diagnostic performance of CCTA.

Using anatomical stenosis >50% as the reference standard was the main limitation in the aforementioned studies since an arbitrary cut-off value does not necessarily correlate well with the presence of myocardial ischemia. Moreover, all studies comparing diagnostic accuracy between patients with and without DM suffer from selection bias as they only included patients referred for ICA. Subsequently, it could be hypothesized that diagnostic accuracy is higher in patients referred for noninvasive testing since the pre-test probability is lower in this population. Moreover, studies were previously performed on CT scanners, which are now considered outdated. The studies discussed provide a nuanced understanding of these differences. However, further prospective research using more advanced imaging technology is needed to fully elucidate the impact of DM on the diagnostic performance of CCTA.

In summary, several factors suggest that the diagnostic accuracy of CCTA for detecting obstructive CAD may vary between patients with and without DM. Patients with DM tend to have a higher pre-test probability of CAD, and the characteristics of atherosclerotic disease, such as higher plaque burden and greater calcification, differ from individuals without DM. These factors can reduce the specificity of CCTA in patients with DM, particularly as the CACS increases. While some studies have shown no significant difference in diagnostic accuracy between patients with and without DM [30, 31], one study reported significantly lower accuracy in patients with DM, likely due to differences in the CACS [29].

### Factors Influencing Diagnostic Accuracy of CCTA in Patients with DM

Several key factors influence the diagnostic accuracy of CCTA, which can broadly be classified as patient-, CT-scan equipment- and post-processing-related. Several patient-related factors have been associated with low image quality; as discussed, calcified lesions can reduce diagnostic accuracy. Another important patient-related factor during the CCTA is heart rate. Several studies have shown that higher heart rates and greater heart rate variability negatively affect image quality, thereby impairing the diagnostic accuracy of CCTA for detecting obstructive CAD [32, 33, 34]. This is particularly relevant in patients with DM, as cardiovascular autonomic neuropathy, which causes autonomic dysregulation, may lead to a higher resting heart rate in these individuals [35]. Consequently, patients should be premedicated with negative chronotropic agents, such as beta-blockers, to lower heart rate and variability by suppressing ectopy. Obesity is widely regarded as the most significant risk factor for DM, with 80–90% of individuals with T2DM being overweight [36]. Meanwhile, a lower diagnostic accuracy in diagnosing obstructive CAD in patients with obesity has been reported [37].

In recent years, many technical modifications have been developed, leading to an increase in CT quality. Advancements, including enhanced software for image acquisition and post-processing, dual-source scanners, spectral CT detectors, and photon-counting CT detectors, can potentially counteract the reductions in diagnostic accuracy caused by the aforementioned patient-related factors, thereby enhancing diagnostic accuracy in patients with DM. These innovations have significantly mitigated motion artifacts associated with elevated heart rates and variability, reduced calcium blooming, and enhanced spatial resolution [38]. These have been further augmented by the development of faster gantry rotation times and advanced electrocardiogram (ECG) gating techniques, which synchronize image acquisition with the cardiac cycle. Innovations in image reconstruction algorithms, such as iterative reconstruction and artificial intelligence-based methods, have enhanced image clarity and reduced noise, contributing to better spatial and temporal resolution [39]. Andreini *et al*. (2018) [40] investigated the diagnostic accuracy of coronary CCTA in a cohort of 202 patients, comparing those with a heart rate above 80 beats per minute (bpm) to those with a heart rate below 65 bpm. Utilizing a dual-source 320-detector row CT scanner, the study incorporated iterative reconstruction techniques and an intra-cycle motion correction algorithm to enhance image quality and diagnostic precision. The mean image quality, assessed using a 4-point Likert scale, was high in both patient groups (3.35 *vs*. 3.39, respectively), similar to coronary interpretability per segment (97.3% *vs*. 98%, respectively). In patients with a heart rate >80 bpm, sensitivity and specificity for detecting >50% stenosis on the ICA were 95.2% and 98.8%, respectively [40]. Mangold and colleagues (2017) [41] retrospectively investigated the diagnostic accuracy in 76 patients, of which 39 had a BMI ≥30 kg/m^2^ and underwent CCTA and ICA. A third-generation dual-source CT with automated tube voltage selection and ECG-triggered acquisition was used. Sensitivity and specificity were reported at 96.6% and 95.5%, respectively. No significant difference between obese and non-obese patients was observed [41]. These advancements for patients with DM, who often present with complex cardiovascular profiles, mean that CT imaging is now a more reliable tool for detecting CAD. The enhanced imaging accuracy, regardless of heart rate or obesity, makes next-generation CCTA an increasingly suitable test for patients with DM.

Furthermore, photon-counting detectors have greatly increased the spatial resolution of CCTA [42]. In a study by Halfmann and colleagues [43], the impact of high (0.4 mm) and ultrahigh (0.2 mm) spatial resolution was assessed and compared to standard (0.6 mm) resolution using photon-counting detector CT, both *in vitro* and *in vivo*. The *in vitro* analysis with simulated calcified lesions at 25% and 50% stenosis revealed significantly reduced overestimation at higher resolutions: for 25% stenosis, with overestimations at 10.7% (0.6 mm), 8.5% (0.4 mm), and 2.0% (0.2 mm) (*p* < 0.001); for 50% stenosis, overestimations were 9.5% (0.6 mm), 7.5% (0.4 mm), and 2.7% (0.2 mm) (*p* < 0.001). A total of 114 patients demonstrated a significant reduction (*p* < 0.01) in diameter stenosis percentage with ultrahigh (29.2% [interquartile range (IQR) 18.3–47.1%]) and high resolution (36.7% [IQR 23.7%–55.1%]) in the* in vivo* results, compared to standard resolution (41.5% [IQR 27.2%–59.2%]) [43]. A prospective study in which 68 patients were included evaluated the diagnostic accuracy of photon counting detector CT, using ICA (stenosis >50%) as the reference standard. A sensitivity of 96%, specificity of 84%, and accuracy of 88% were reported in this study [44]. These relatively small studies show promising results using the photon-counting detector CT in high-risk patients with severe calcifications. However, prospective studies evaluating potential advances in diagnostic accuracy in patients with DM are warranted.

Various post-processing tools have been developed to enhance the diagnostic accuracy of coronary CCTA. The application of one such tool, CT-FFR, as derived by computational fluid dynamics (CFD), results in the augmentation of diagnostic precision [45, 46, 47]. A post hoc study of the PACIFIC (The Prospective Comparison of Cardiac PET/CT, SPECT/CT Perfusion Imaging and CT Coronary Angiography With Invasive Coronary Angiography) trial evaluated the incremental value of CT-FFR in conjunction with CCTA for diagnosing ischemia-inducing lesions in 208 patients with suspected stable CAD. The study showed that CT-FFR significantly improved the area under the curve for detecting ischemia-causing lesions compared to CCTA alone, both on a per-vessel basis (0.94 *vs*. 0.83, *p* < 0.001) and a per-patient basis (0.92 *vs*. 0.81, *p* = 0.002) [48]. The ADVANCE (Assessing Diagnostic Value of Noninvasive CT-FFR in Coronary Care) registry evaluated 4290 patients, of which 942 (22%) with DM underwent CCTA for suspected CAD with at least one stenosis greater than 30% and received CT-FFR analysis. A total of 3900 (72.4%) patients showed obstructive CAD (≥50% stenosis in any vessel), and 2868 (66.9%) had a positive CT-FFR result (≤0.80). Patients with DM were more likely to show obstructive CAD on the CCTA than those without DM after adjusting for demographics and risk factors (78.8% *vs*. 70.6%, *p* < 0.001). Moreover, patients with DM were more likely to have a positive CT-FFR (74.3% *vs*. 64.8%, *p* < 0.001) and were more likely to have positive CT-FFR findings in multiple vessels. However, the presence of DM did not lead to differences in treatment strategies. When stratifying for CT-FFR positivity and stenosis severity, no difference in MACEs was observed between patients with and without DM at the 1-year follow-up. These findings suggest that a CCTA plus CT-FFR strategy yields comparable outcomes in patients with DM to those without, making it an appropriate diagnostic approach. However, it is important to note that CCTA plus CT-FFR was not compared directly to ICA, meaning the diagnostic accuracy relative to ICA remains unassessed. Additionally, more data, specifically from patients with DM, are needed, as the proportion of patients with DM in this registry was relatively small [49]. Furthermore, dynamic CT myocardial perfusion imaging (MPI) allows ischemia to be assessed. Yu and colleagues compared CT myocardial perfusion imaging (CT-MPI) [50] in addition to CCTA to CCTA alone in 240 patients (mean age 69, 67% men, 21.7% DM) with chest pain and intermediate likelihood of CAD. Interestingly, patients were less likely to undergo ICA in the CT-MPI group compared to the CCTA group (30.8% *vs*. 48.3%, respectively; *p* = 0.006) and ICA without revascularization (10.8% *vs*. 50%, respectively, *p* < 0.0001) [50]. However, whether the test result is clinically useful in patients with DM remains to be investigated.

Thus, several patient-related factors influence the diagnostic accuracy of CCTA in individuals with DM, including calcified lesions and higher resting heart rates due to cardiovascular autonomic neuropathy, which can negatively impact image quality. Obesity, common in patients with T2DM, is also associated with lower CCTA accuracy. Recent advancements in CT technology, such as dual-source scanners and photon-counting detectors, have improved imaging quality by addressing motion artifacts and calcified plaque overestimation. These innovations and post-processing tools, such as CT-FFR, have enhanced diagnostic accuracy, particularly for detecting ischemia-inducing lesions. Studies show promising results, suggesting that next-generation CCTA is a reliable tool for detecting CAD in patients with DM. However, further prospective studies are needed to assess these advancements fully in patients with DM.

## 3. CCTA as a Key Tool in Enhancing Cardiovascular Outcomes and Guiding Medical Management in Patients with DM and Stable Chest Pain

### 3.1 Impact of Initial Diagnostic Testing Strategy on Cardiovascular Outcomes

Emerging evidence suggests that using CCTA as a diagnostic test can potentially improve cardiovascular outcomes in patients with DM and chest pain (Table 2, Ref. [7, 8, 51, 52, 53]). The SCOT-HEART (Scottish Computed Tomography of the Heart) trial, which included 4146 patients (mean age 57.1 years, 56% male and 11% diagnosed with DM), compared the standard-of-care plus CCTA to the standard-of-care alone. These results demonstrated a significant reduction in the composite primary outcome of non-fatal myocardial infarction and death due to coronary heart disease (2.3% *vs*. 3.9%, *p* = 0.004) in favor of the CCTA group at the 5-year follow-up (hazard ratio (HR) 0.59 [95% CI 0.41–0.84]). This reduction was even more pronounced in the subpopulation with DM, with an absolute risk reduction more than three times greater than in patients without DM (4.6% *vs*. 1.3%) [7]. In the PROMISE (Prospective Multicenter Imaging Study For Evaluation of Chest Pain) trial, which enrolled 10,003 patients (mean age 60.8 years, 52.7% female and 21.4% diagnosed with DM) with stable chest pain and an intermediate pre-test probability of CAD, no significant difference was found in the primary composite outcome between CCTA and functional testing during a median follow-up of 25 months (3.3% *vs*. 3.0%, *p* = 0.75). However, a significant reduction in MACEs was observed in the subgroup with DM undergoing CCTA compared to those undergoing functional testing (1.1% *vs*. 2.6%) [8, 51]. The DISCHARGE (Diagnostic Imaging Strategies for Patients with Stable Chest Pain and Intermediate Risk of Coronary Artery Disease) trial compared CCTA with ICA in 3561 patients (mean age 60.1 years, 56.2% female and 15.6% diagnosed with DM) over a median follow-up of 3.5 years. The trial demonstrated that CCTA had comparable safety and efficacy to ICA in the overall population, with fewer major procedure-related complications (0.5% *vs*. 1.9%) and revascularizations (14.2% *vs*. 18.0%). There was no significant difference in the incidence of major MACEs between the CCTA group (2.1%) and the ICA group (3.0%) (HR 0.70; 95% CI 0.46–1.07; *p* = 0.10). In a subgroup analysis where patients with DM were evaluated, the incidence of MACEs was 3.8% in the CCTA group compared to 6.5% in the ICA group (HR 0.58 [95% CI, 0.27–1.25], *p* = 0.45). Additionally, procedure-related complications were less frequent in patients with DM undergoing CCTA (0.4%) versus those undergoing ICA (2.7%) (HR 0.30, [95% CI 0.13–0.63]) [52, 53].

**Table 2.** **Randomized controlled trials evaluating CCTA as an initial diagnostic test in chest pain evaluation**.

Trial	Sample size	No. of patients with diabetes (%)	Intervention	Comparison	Median follow-up	Primary endpoint	Primary outcome (all patients)	Primary outcome (subpopulation of patients with diabetes)
SCOT-HEART [7]	4146	444 (11%)	CCTA + SOC	SOC	4.8 years	MACEs (non-fatal myocardial infarction and death due to CAD)	2.3% in CCTA group *vs*. 3.9% in SOC group (HR 0.59 [95% CI 0.41–0.84], *p* = 0.004)	3.1% in CCTA group *vs*. 7.7% in SOC group (HR 0.36 [95% CI 0.15–0.87])
PROMISE [8, 51]	10,003	2144 (21.4%)	CCTA	Functional testing	25 months	MACEs (death, myocardial infarction, hospitalization for unstable angina, major procedural complications)	3.3% in CCTA group *vs*. 3.0% in functional testing group (HR 1.04 [95% CI 0.83–1.29], *p* = 0.75)	1.1% in CCTA group *vs*. 2.6% in functional testing group (HR 0.38 [95% CI 0.18–0.79])
DISCHARGE [52, 53]	3561	557 (15.6%)	CCTA	ICA	3.5 years	MACEs (cardiovascular death, non-fatal myocardial infarction, non-fatal stroke)	2.1% in CCTA group *vs*. 3.0% in ICA group (HR 0.70 [95% CI 0.46–1.07], *p* = 0.10)	3.8% in CCTA group *vs*. 6.5% in ICA (HR 0.58 [95% CI 0.27–1.25])

Abbreviations: CCTA, coronary computed tomography angiography; ICA, invasive coronary angiography; MACEs, major adverse cardiac events; HR, hazard ratio; CI, confidence interval; SOC, standard-of-care; CAD, coronary artery disease.

The observed reduction in cardiovascular events within the CCTA group is likely due to the more frequent diagnosis of CAD, which resulted in a higher rate of initiation of preventive medical therapy (odds ratio (OR) for initiating preventive medical therapy 1.40 [95% CI, 1.19–4.65]) in the SCOT-HEART trial. After 5 years, 59% of patients in the CCTA group received statin therapy compared to 50.3% in the standard-of-care (SOC) group. Additionally, a larger proportion of patients in the CCTA group were on antiplatelet therapy (50.6% *vs*. 40.5%, respectively) [7].

### 3.2 Optimizing Preventive Medical Therapy in Patients with DM and CAD

**Fig. 1.**
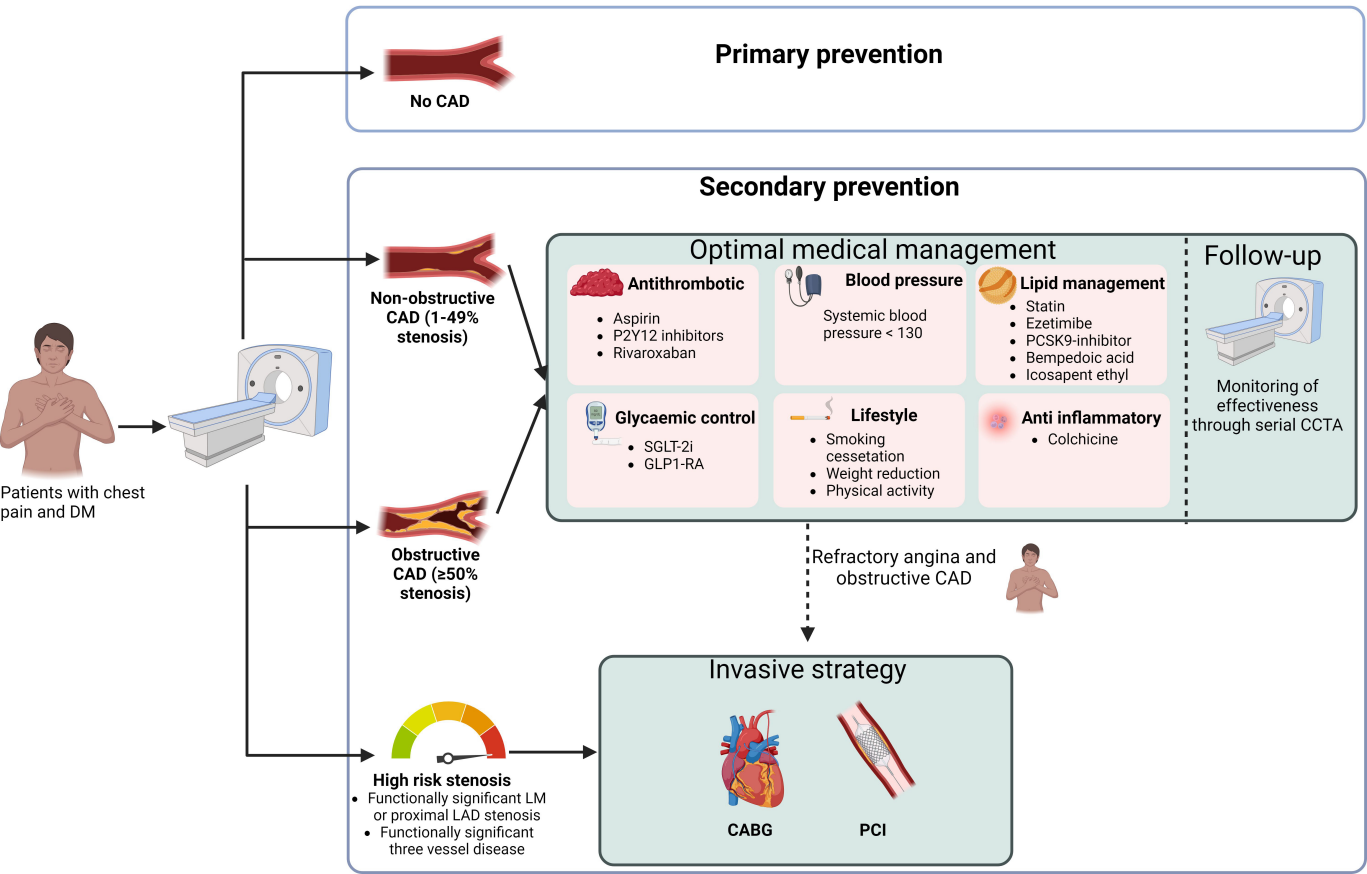
**Management of patients with diabetes mellitus and chest pain using CCTA as the initial diagnostic test**. DM, diabetes mellitus; CAD, coronary artery disease; LM, left main; LAD, left anterior descending artery; SGLT-2i, sodium–glucose transport protein 2 inhibitor; GLP1-RA, glucagon-like peptide-1 receptor agonist; PCSK9 inhibitor, proprotein convertase subtilisin/kexin type 9 inhibitor; CCTA, coronary computed tomography angiography; CABG, coronary artery bypass grafting; PCI, percutaneous coronary intervention. Figure created using BioRender.com.

In the SCOT-HEART trial, 654 (37%) patients presented with non-obstructive CAD [7]. Identifying these patients allows for adequate prescription of preventive medication in patients with DM and non-obstructive CAD [5]. The Swedish National Diabetes Register matched 271,174 patients with T2DM to 1,355,870 controls based on age, sex, and country. Five risk factors were assessed: hypercholesterolemia, albuminuria, smoking, hypertension, and elevated glycated hemoglobin level. The results showed that patients with T2DM and all five risk factor variables within the target range had a similar risk of death from any cause (HR 1.06 [95% CI 1.00–1.12]) and stroke (0.95 [0.84–1.07]) when compared to patients without T2DM. Meanwhile, the risk of myocardial infarction was even slightly lower in patients with T2DM (HR 0.84 [0.75–0.93]) [54]. These findings highlight the potential for patients with T2DM to achieve comparable health outcomes to individuals without T2DM through rigorous management of these risk factors, emphasizing the importance of comprehensive risk factor control in this population. The importance of optimal preventive medical therapy has also been demonstrated in patients with obstructive CAD and DM. The ISCHEMIA (International Study of Comparative Health Effectiveness with Medical and Invasive Approaches) randomized controlled trial of 5179 patients investigated whether patients with moderate to severe ischemia, determined by stress testing, benefit from an initial invasive strategy (ICA and revascularization if feasible) compared to an initial conservative strategy. Patients underwent CCTA to exclude left main stenosis or non-obstructive CAD. No difference was observed in the primary endpoints of cardiovascular death, myocardial infarction, hospitalization for unstable angina, heart failure, or resuscitated cardiac arrest (HR 0.93 [95% CI 0.80–1.08], *p* = 0.34) after a median follow-up of 3.2 years; there was also no difference in patients with DM (HR 0.92 [95% CI 0.74–1.15]) [55]. Similar results were found in patients with T2DM in the BARI 2D (Bypass Angioplasty Revascularization Investigation 2 Diabetes) randomized controlled trial. In this trial, 2368 patients with T2DM were assigned to initial revascularization or initial medical management, while no significant differences were noted in the survival rates (88.3 *vs*. 87.8, respectively; *p* = 0.97) at 5 years [56]. These findings advocate for a primary conservative management strategy in patients with stable CAD, with revascularization reserved for cases where angina proves refractory to optimal medical therapy (Fig. 1).

In recent years, the introduction of several lipid-lowering therapies, including proprotein convertase subtilisin/kexin type 9 (PCSK9) inhibitors, inclisiran, and icosapent ethyl, has broadened treatment options [57, 58]. Furthermore, glucagon-like peptide-1 receptor agonists (GLP1-RAs) and sodium–glucose cotransporter-2 inhibitors (SGLT-2is) have effectively reduced cardiovascular events in patients with DM [59, 60]. Additionally, therapies targeting the proinflammatory and prothrombotic states in patients with DM, such as low-dose rivaroxaban and colchicine, have proven beneficial [61, 62]. Given the high costs associated with these therapies, it is essential to carefully select patients at the highest risk of cardiovascular events to ensure that those who could benefit most are appropriately identified and treated (Fig. 1).

### 3.3 Screening for CAD in Asymptomatic Patients with DM Using CCTA

The CONFIRM registry (Coronary CT Angiography Evaluation for Clinical Outcomes: An International Multicenter Registry) included 23,643 patients without a prior history of CAD who underwent CCTA. From this cohort, 3370 patients with DM were propensity matched with 6740 patients without DM. The findings revealed that individuals with DM were less likely to have normal coronary arteries, indicating no atherosclerosis than those without DM (28% *vs*. 36%, *p* < 0.0001). Additionally, patients with DM had a higher prevalence of obstructive CAD compared to patients without DM (37% *vs*. 27%). The prevalence of asymptomatic patients was higher amongst patients with DM (34% *vs*. 30%, *p* = 0.0006) [63]. At the 5-year follow-up in this study, patients with DM and non-obstructive CAD had an increased risk of all-cause mortality compared to patients without DM (HR 2.10 [95% CI 1.43–3.09], *p* < 0.001). This difference was not observed in patients without atherosclerotic plaques (HR 1.32 [95% CI 0.78–2.24], *p* = 0.296) [64]. The FACTOR-64 (Effect of Screening for CAD Using Computed Tomography Angiography on Mortality and Cardiac Events in High-Risk Patients with Diabetes) trial is the only randomized study to date that has evaluated the impact of using CCTA for screening asymptomatic patients with DM on cardiovascular outcomes. In this trial, 900 asymptomatic patients with DM were randomized to CAD screening using CCTA or standard DM care, with a median follow-up duration of 4 years. No significant difference was observed for the composite primary endpoints consisting of all-cause mortality, non-fatal myocardial infarction, or unstable angina requiring hospitalization between the CCTA group and the standard-of-care group (HR 0.80 [95% CI 0.49–1.32], *p* = 0.38) [65]. Enrollment in the FACTOR 64 trial occurred before the availability of GLP1-RA and SGLT-2i, which have been shown to be effective in reducing cardiovascular events in patients with established CAD [60, 66]. Furthermore, the evaluation of significant stenosis was the main focus of CCTA assessment rather than the presence of atherosclerotic plaques. Future clinical studies should further evaluate the effectiveness of screening for CAD in asymptomatic patients, guiding drug therapy and, thus, reducing cardiovascular events.

## 4. Future Perspectives on the Clinical Implications of CCTA in Patients with DM

### 4.1 CCTA for Risk Stratification in Patients with Stable Chest Pain and DM

**Fig. 2.**
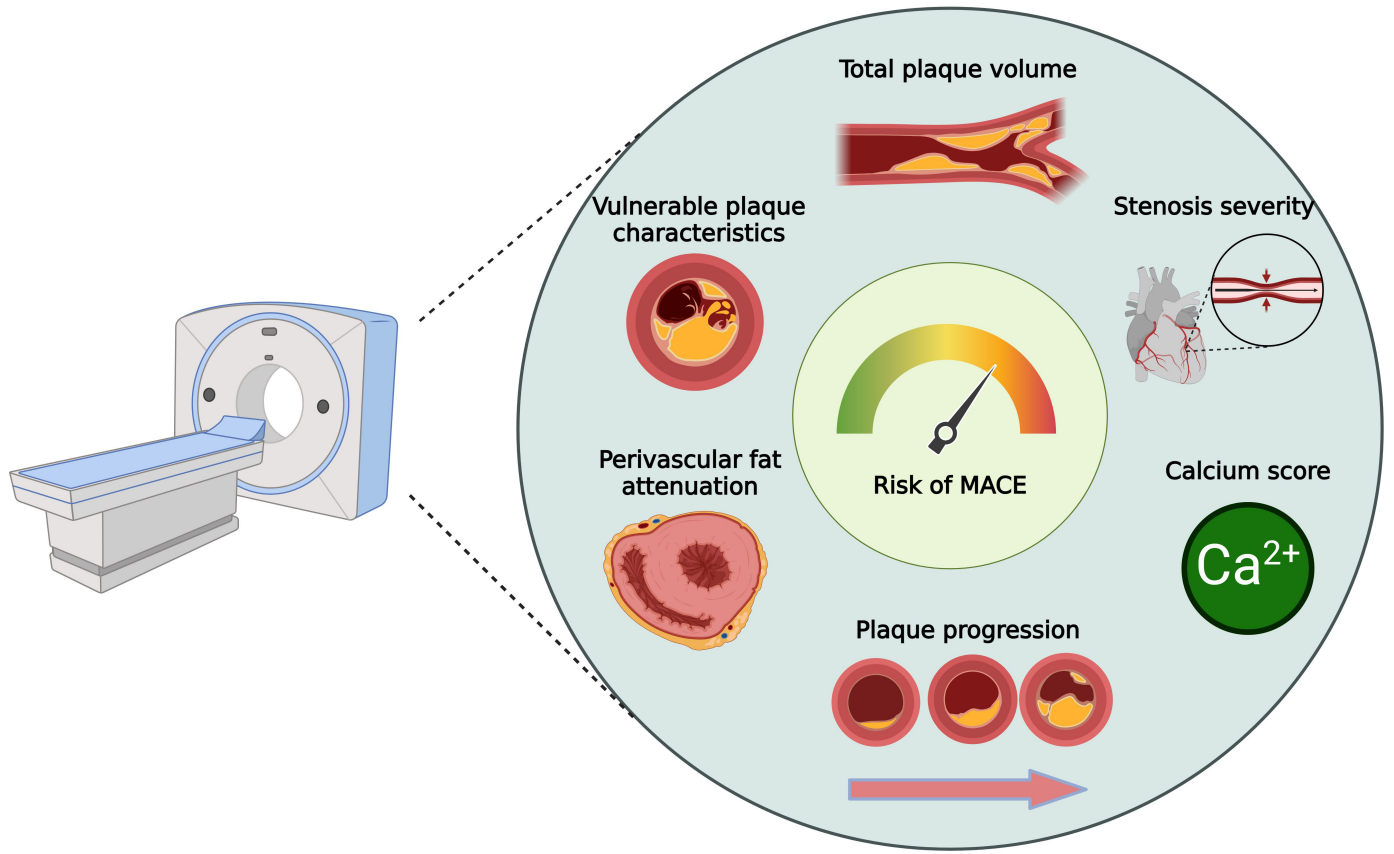
**CCTA biomarkers for personalized risk assessment in patients with diabetes mellitus**. MACE, major adverse cardiovascular event; CCTA, coronary computed tomography angiography. Figure created using BioRender.com.

As previously discussed, CCTA offers comprehensive insights for diagnosing obstructive CAD beyond assessing lumen stenosis (Fig. 2). CCTA also facilitates the detailed examination of vessel walls, allowing for both qualitative and quantitative assessments of CAD. Moreover, high-risk features discerned through CCTA can be instrumental in identifying vulnerable plaques. Indeed, vulnerable plaques, marked by their tendency for rapid progression and/or rupturing, play a pivotal role in the occurrence of coronary events [67, 68]. Thin cap fibroatheromas, which are plaques prone to rupturing, are histopathologically characterized by a thin, inflamed fibrous cap, a necrotic lipid-rich core, and the presence of spotty- and microcalcifications [69, 70, 71]. Several plaque characteristics observable on the CCTA are associated with thin cap fibroatheromas, such as positive remodeling, low attenuation plaques, spotty calcifications, and the napkin-ring sign [72, 73, 74]. Post hoc analyses of the SCOT-HEART and PROMISE trials have shown that high-risk plaque characteristics are associated with incident MACEs in patients with stable chest pain [15, 16]. Within the PROMISE trial, patients with high-risk plaques (positive remodeling, low CT attenuation, or napkin-ring sign) had an increased risk of MACEs compared to patients without high-risk plaques (6.4% *vs*. 2.4%, respectively, HR 2.73 [95% CI 1.89–3.93]). The addition of high-risk plaques to a model that included significant stenosis and atherosclerotic cardiovascular disease (ASCVD) risk score (Framingham heart study risk score) led to a net reclassification improvement (0.34 [95% CI 0.02–0.51]) [15]. In a post hoc analysis of the SCOT-HEART study, high-risk plaque features (low attenuation, spotty calcification, positive remodeling, napkin-ring sign) were assessed in 1769 patients and were present in 608 (34%). Death from coronary heart disease and non-fatal infarction were more frequent in patients with high-risk plaque features than patients without (4.1% *vs*. 1.4%, respectively, HR 3.01 [95% CI 1.61–5.63], *p* = 0.001). There was no difference in the risk for adverse events between patients with and without high-risk plaque characteristics when the CACS exceeded 100 AU [16]. Meanwhile, plaque burden and plaque progression have also been shown to be predictors of cardiovascular events. Lin *et al*. (2022) [75] showed that a total plaque burden above 238.5 mm^3^ is associated with an increased risk of fatal or non-fatal myocardial infarction in 1611 patients from the SCOT-HEART trial (HR 5.36 [95% CI 1.70–16.86], *p* = 0.0042). The PARADIGM (Progression of Atherosclerotic Plaque Determined by Computed Tomographic Angiography Imaging) study included 1166 patients who underwent serial CCTA because of stable chest pain, with a median follow-up of 8.2 years; the mean age was 60.5 years, 55% were male, and 22.6% had DM. In this study, an increase in non-calcified plaques was independently associated with MACEs (OR 1.23 [95% CI 1.08–1.39] per increase of one standard deviation) during a median follow-up of 8.2 years [76]. DM was an independent risk factor for plaque progression (OR 1.53 [95% CI 1.10–2.12], *p* = 0.011). Furthermore, the frequency of high-risk plaque features was significantly higher in patients with DM [77]. Jonas *et al*. (2023) [17] compared atherosclerotic plaque characteristics observed by CCTA between patients with and without DM in 303 patients, of which 95 patients with DM were referred for ICA. Patients with DM and a non-obstructive disease showed a greater plaque volume, more non-calcified plaques, and higher diseased vessels, although no difference in high-risk plaque characteristics was observed [17]. These studies provide insights into the natural history of CAD in patients with DM and highlight that differences in plaque characteristics were associated with an elevated risk of MACEs. Integrating CCTA into routine clinical practice for patients with DM with stable chest pain could refine the current approach to risk evaluation and therapeutic decision-making; however, more robust data are needed. By distinguishing between patients likely to benefit from specific medical interventions and those who do not, CCTA can help optimize treatment strategies, potentially enhancing patient outcomes. Moreover, serial CCTA imaging could provide valuable feedback on the effectiveness of medical therapies in stabilizing plaques and reducing cardiovascular risk. Zhang and colleagues [78] recently highlighted the impact of SGLT-2is on atherosclerotic plaque progression in 236 patients with T2DM, 134 of whom were treated with SGLT-2is. Patients who underwent at least 2 CCTA examinations with a minimum interval of 12 months were included. After a median treatment duration of 14.6 months, the study demonstrated a significant reduction in non-calcified and low-attenuation plaque volumes, while calcified plaque volume significantly increased [78]. In another study involving 204 asymptomatic patients with T2DM, 55 of whom (27%) were treated with liraglutide, a GLP1-RA, a greater increase in fibrous plaque volume was observed in the liraglutide-treated group after a median scan interval of 13 months (*p* = 0.04). However, there was no change in the total atheroma volume [79]. These studies offer valuable insights into the mechanisms underlying risk reduction in patients with DM. Moreover, these studies highlight the promising potential of using serial CCTA to monitor therapeutic responses in this patient population.

The pericoronary fat attenuation index (FAI) is a novel imaging biomarker derived from CCTA, which quantifies the inflammatory burden around coronary arteries by measuring the attenuation of perivascular adipose tissue (PVAT) [80, 81]. The ORFAN (Oxford Risk Factor And Non-invasive Imaging) study evaluated the prognostic value of the FAI in 3393 patients with obstructive and non-obstructive CAD. During a median follow-up period of 2.7 years, an increased FAI score in all three coronary arteries was associated with MACEs (HR 12.6 [95% CI 8.5–18.6]) [82]. Ichikawa and colleagues (2022) [83], who evaluated the prognostic value of the FAI in 333 patients with T2DM, found a significantly higher FAI of the left anterior descending artery in patients who had a cardiovascular event (–68.5 ± 6.5 HU *vs*. –70.8 ± 6.1 HU, *p* = 0.045). However, there was no association between the FAI of the right coronary artery and cardiovascular events (*p* = 0.09) [83]. A model using both the FAI and low attenuation plaque burden predicted myocardial infarction in a post hoc analysis of 1697 patients in the SCOT-HEART trial (HR 11.7, *p* < 0.0001) [84]. The FAI can potentially improve risk stratification in CAD and DM patients.

### 4.2 Artificial Intelligence in CCTA Analyses

Implementing artificial intelligence (AI) into CCTA analyses to quantify calcified and non-calcified plaques and the degree of coronary stenoses enhances the efficiency, accuracy, and reproducibility of diagnosing CAD [85, 86, 87, 88]. Traditional analysis of the CCTA relies on the visual estimation of the stenosis severity, subject to significant interobserver variability and biases, which AI-powered algorithms reduce [86]. For example, Choudhary *et al*. (2011) [89] found interobserver reliability for stenosis severity varies between segments, endorsing the need for more consistent methods. Furthermore, AI-powered algorithms provide reproducible assessment unaffected by intra- and interobserver variability, leading to more reliable diagnoses and treatment plans [86].

Several AI-based methods have been described for the automatic CAD-RADS (coronary artery disease-reporting and data system) score prediction, of which the performance nearly reached an interobserver agreement [75, 90, 91]. Lin *et al*. (2022) [75] externally validated a deep learning-based automatic CAD-RADS algorithm, initially trained on a cohort of 921 patients. This validation was performed on an independent cohort of 175 patients. Across the entire study population, including the training and validation cohorts, the mean age was 65.2 years, 65.2% were male, and 18.5% had DM. The agreement between the expert assessments and the deep learning CAD-RADS algorithm was 87% [75].

Recent advancements in AI-augmented software allow for automated quantitative plaque analysis, facilitating reproducible measurements of plaque burden, differentiation between calcified and non-calcified plaques, and the detection of high-risk plaque features [92]. Within the CLARIFY (CT Evaluation by Artificial Intelligence For Atherosclerosis, Stenosis, and Vascular Morphology) study, high-risk features defined as positive remodeling and low attenuation were predicted in 232 patients undergoing CCTA; the mean age was 60 years, 37% were female, and 29% were diagnosed with DM. Consensus between the convolutional neural network and the expert panel was measured with a kappa statistic of 0.372, indicating moderate agreement despite reasonable disagreement among the experts [91]. A recent publication detailed the external validation of a promising AI algorithm to predict MACEs. This prognostic tool integrates coronary plaque characteristics, clinical risk factors, and the FAI. The validation cohort included 46.4% males, with a median age of 62 years, and 16.3% had a diagnosis of DM. The AI classification demonstrated a significant association with MACEs when comparing high-risk patients to patients with a medium or low risk (HR 4.68 [95% CI 3.93–5.57], *p* < 0.001) [82].

While CCTA primarily evaluates the morphological characteristics of coronary plaques, recent advances in AI-based methods for CCTA assessment are increasingly enabling the evaluation of the hemodynamic significance of coronary stenosis [93, 94, 95, 96]. As mentioned, calculating the CT-FFR using the CFD has improved the diagnostic accuracy of CCTA in identifying lesions that potentially cause ischemia. CFD simulations of coronary arterial flow from CCTA images are complex and computationally inefficient due to the need to accurately model both heart and arterial dynamics. Deep learning approaches can overcome these limitations by quickly learning and predicting the FFR without detailed physical modeling, making the process faster and more efficient [97]. In a multicenter trial involving 351 patients, Coenen *et al*. (2018) [96] compared the diagnostic performance of machine learning (ML)-based CT-FFR with CFD-based CT-FFR for detecting hemodynamically significant CAD, using invasive FFR as the reference standard. The cohort had a mean age of 62.3 years, while 74% were male, and 21% had DM. Patients were recruited prospectively from one site and retrospectively from four sites based on the availability of CCTA and invasive FFR data. The results showed an excellent correlation between ML-FFR and CFD (R = 0.997). In patients with DM, the overall diagnostic accuracy of CT-FFR was 83%, compared to 75% in patients without DM (*p* = 0.088). However, CT-FFR demonstrated significant improvement over coronary CT angiography alone, which had accuracies of 58% and 65% (*p* = 0.223), respectively [96, 98]. In addition to DL-derived CT-FFR, other AI-based methods have been developed to identify hemodynamically significant stenoses. Hampe *et al*. (2022) [95] employed a convolutional neural network to predict lumen area, average lumen attenuation, and calcium area from multiplanar reconstructions in a cohort of 569 patients. The study reported an area under the curve of 0.78 for predicting the presence of functionally significant stenoses, using invasively measured FFR as the reference standard [95].

**Fig. 3.**
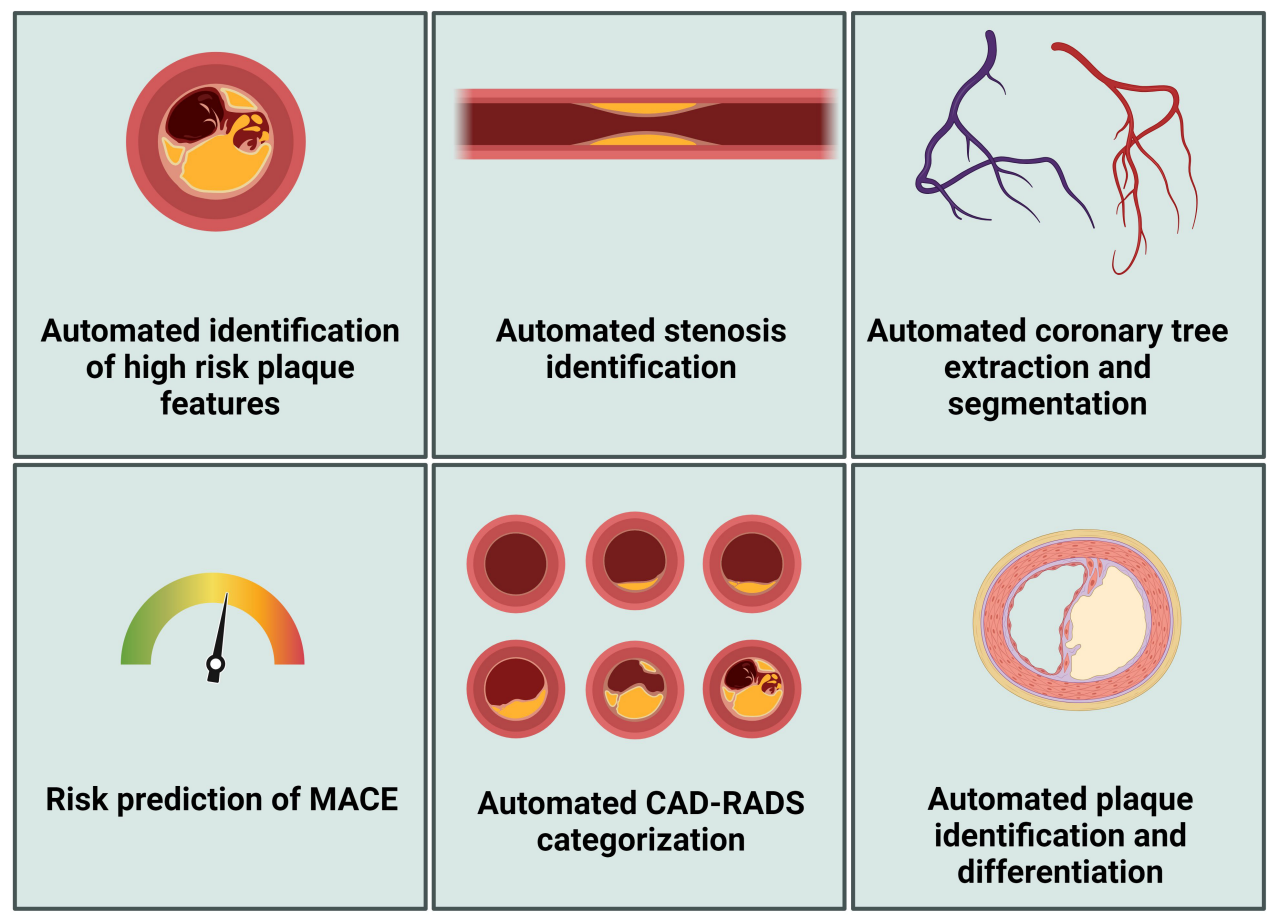
**Artificial intelligence applications in CCTA analysis**. MACE, major adverse cardiovascular event; CCTA, coronary computed tomography angiography; CAD-RADS-score, coronary artery disease-reporting and data system score. Figure created using BioRender.com.

Various research and clinical applications have been developed using AI for assessing coronary artery plaques (Fig. 3), and relevant tools have recently been identified and summarized by Föllmer *et al*. (2024) [88]. For example, Cleerly (Denver, Co) developed Food and Drug Administration-approved software that can be used for the segmentation and labeling of coronary arteries, identification of images of good quality, and identification of the vessel wall [99]. Further, Heartflow Analysis provides automatic plaque detection and characterization for risk assessment [100]. Föllmer *et al*. (2024) [88] concluded that the proprietary technologies behind these commercial products are often inaccessible to users, potentially leading to inconsistencies in their clinical application. Additionally, Föllmer *et al*. (2024) [88] noted that many scientific publications do not offer access to source code, data, or trained models in public repositories, which impedes reproducibility and independent research validation.

The successful application of AI in medical imaging for cardiovascular diseases faces several challenges, with a significant issue being the scarcity of accurately labeled data. Training effective AI models necessitate large volumes of high-quality labeled data, which is labor-intensive and requires the expertise of experienced physicians. The data quality is particularly crucial, as inconsistencies or inaccuracies can impede the performance of AI models. Another challenge is the integration of multimodal imaging and diverse input data, which can enhance outcomes by combining various quantitative imaging and clinical data, thereby offering personalized risk stratification for CAD patients. This approach could aid in identifying high-risk patients and guiding treatment decisions more effectively. Finally, the lack of standardization remains a major obstacle. Indeed, variations in CAD diagnostic standards across different medical institutions and countries complicate efforts to unify data quality and labeling standards, hindering the widespread adoption of AI solutions in this field. Variability in imaging protocols and patient demographics can also affect the performance of AI algorithms, necessitating large and diverse datasets. Privacy concerns are another challenge, as training AI-based algorithms requires patient data sharing. Therefore, data encryption and clear medical ethics guidelines are needed to ensure patient confidentiality and optimal data usage.

Overall, more extensive evaluation studies are needed to validate the clinical impact of AI algorithms, comparing AI-assisted care with standard care to determine the actual benefits in patient outcomes.

## 5. Conclusions

In conclusion, the use of CCTA in the context of patients with DM is rapidly evolving. CCTA enables detailed anatomical visualization of the coronary arteries, including assessing plaque characteristics, total plaque burden, plaque progression, and epicardial fat. A deeper understanding of the clinical consequences of these characteristics may tailor therapy and hold promise for a more personalized medical approach in the future. The high sensitivity and negative predictive value of CCTA make it an excellent tool for excluding significant CAD in patients with DM; therefore, integrating CCTA into clinical practice can potentially improve patient outcomes and optimize healthcare resource utilization.

Moreover, incorporating AI into CCTA analysis enhances diagnostic precision, efficiency, and reproducibility. AI algorithms can automatically detect and quantify coronary artery stenosis, assess plaque characteristics, and predict MACEs, providing more reliable diagnoses and tailored treatment plans.

Future research should continue to refine CCTA techniques, explore the benefits of advanced imaging modalities, and evaluate the long-term impact of CCTA-guided management on cardiovascular outcomes in patients with DM. Additionally, the potential of CCTA as a screening tool in asymptomatic patients warrants further investigation, as current evidence does not demonstrate a clear benefit to cardiovascular outcomes. As evidence continues to accumulate, CCTA is poised to play an increasingly vital role in the stratification and management of cardiovascular risk in this high-risk population.
